# Assessment of Outcomes of Immediately Loaded Dental Implants in Orofacial Cleft Patients: Protocol for a Single-Arm Clinical Trial

**DOI:** 10.2196/25244

**Published:** 2021-05-05

**Authors:** Rizwana Mallick, Sweta Kale Pisulkar, Srinivas Gosla Reddy

**Affiliations:** 1 Department of Prosthodontics and Crown & Bridge Faculty of Dentistry Jamia Millia Islamia New Delhi India; 2 Sharad Pawar Dental College Datta Meghe Institute of Medical Sciences Deemed to be University Wardha India; 3 GSR Institute of Craniomaxillofacial and Facial Plastic Surgery Hyderabad India

**Keywords:** clinical trial protocols, dental implants, dentistry, immediate dental implant loading, implant-supported dental prosthesis, mouth rehabilitation, oral health, orofacial cleft, quality of life, rehabilitation research, treatment outcome

## Abstract

**Background:**

Orofacial cleft, one of the most common congenital deformities, presents with a plethora of defects, subjecting the patient to a multitude of treatments from a young age. Among the oral hard tissue problems, absence of a maxillary permanent tooth in the cleft region either due to congenital absence or extraction due to compromised prognosis is a common finding. Conventionally, the missing tooth is replaced using a removable or fixed partial denture; however, the treatment modality does not satisfactorily meet patient expectations. The most recent decade has seen increasing use of dental implants in the cleft region; however, the outcome of an immediately loaded dental implant is still elusive for orofacial cleft patients.

**Objective:**

This protocol is for a single-arm clinical trial aimed at determining the treatment outcome of immediately loaded dental implants in patients with a nonsyndromic orofacial cleft.

**Methods:**

Patients meeting the set criteria will be sequentially enrolled until a sample size of 30 dental implants is met and will undergo the proposed treatment according to the predecided protocol. All patients will be followed up at the designated time intervals to record various clinical and radiographic parameters. Implant success will be defined based on the criteria elucidated by Misch et al in the Pisa, Italy Consensus. A quality-of-life assessment questionnaire will also be recorded at the end of patient’s follow-up to determine their acceptance of the treatment.

**Results:**

A total of 30 dental implants will be placed in patients with a nonsyndromic orofacial cleft. Obtained results will be statistically analyzed to determine the treatment outcomes and success.

**Conclusions:**

This study will help determine the feasibility of immediately loaded dental implants in compromised bone sites such as those presented in cleft patients and will help in generating findings that can be used to fill the lacunae currently present in the holistic treatment of cleft patients.

**Trial Registration:**

Clinical Trial Registry of India CTRI/2020/09/027997; http://ctri.nic.in/Clinicaltrials/showallp.php?mid1=47659&EncHid=&userName=dental%20implants

**International Registered Report Identifier (IRRID):**

PRR1-10.2196/25244

## Introduction

### Background

Orofacial cleft is the most common congenital anomaly, with an incidence of 1 in 700 to 1 in 1000 live births across different populations [1]. India reports around 28,600 cleft cases every year with a prevalence of 1.09 in 1000 live births [2]. Cleft can be unilateral or bilateral, occurring either alone or a combination of lip and palate, with or without the involvement of the alveolar process. Complete clefting of the lip that involves the full height is often associated with cleft of the alveolus. In addition to compromised aesthetics and disoriented attachment of musculature leading to compromised functionality, these patients also suffer from various dental anomalies [3]. Tooth agenesis affecting the maxillary lateral incisor in the cleft region is the most commonly found anomaly followed by crowding and delayed development [4,5]. All of these defects in cumulation compromise the patient’s quality of life, and each case poses a challenge for the multidisciplinary health care team due to the unique presentation.

Prosthetic rehabilitation plays a triple role of improving aesthetics, phonetics, and functionality of the patient [6]. Use of dental implants has increasingly become popular, as they help in maintaining the bone dimensions in the reconstructed region along with provision of improved aesthetics compared to conventional replacement options. Dental implant can be opted for only when the patient has reached skeletal maturity so as to avoid potential growth hindrance. Postinsertion, an implant requires a healing period of 3-6 months to firmly integrate with the underlying bone, which is then adequately loaded with a prosthesis. Considering the long treatment duration and compromised aesthetics already incurred by the patient, it is necessary to develop protocols that help reduce the rehabilitation time. Immediate implant loading is one such measure wherein acceptable initial implant stability enables implant loading in as little time as 1 week. This would not only decrease time lapse in 2 consecutive rehabilitative procedures but also help improve the patient’s psychological acceptance. Most studies presented have performed implant loading after the universally followed protocol of 6 months [7], while a few studies have also demonstrated loading after 3 months [8]. Only 1 study has been conducted so far that presented the results of immediate or early implant loading, but it was a retrospective analysis [8,9].

### Aim

The aim of this study is to evaluate the clinical and radiographic success of immediately loaded dental implants in patients with an orofacial cleft.

### Objectives

The objectives of the study are to evaluate the placed single dental implant in the cleft region for clinical and radiological parameters 3 months after dental implant placement; evaluate the placed single dental implant in the cleft region for clinical and radiological parameters 9 months after dental implant placement (6 months after definitive prosthesis); and evaluate and compare the placed single dental implant in the cleft region for clinical and radiological parameters 3 months and 9 months after dental implant placement.

## Methods

**Figure 1:**
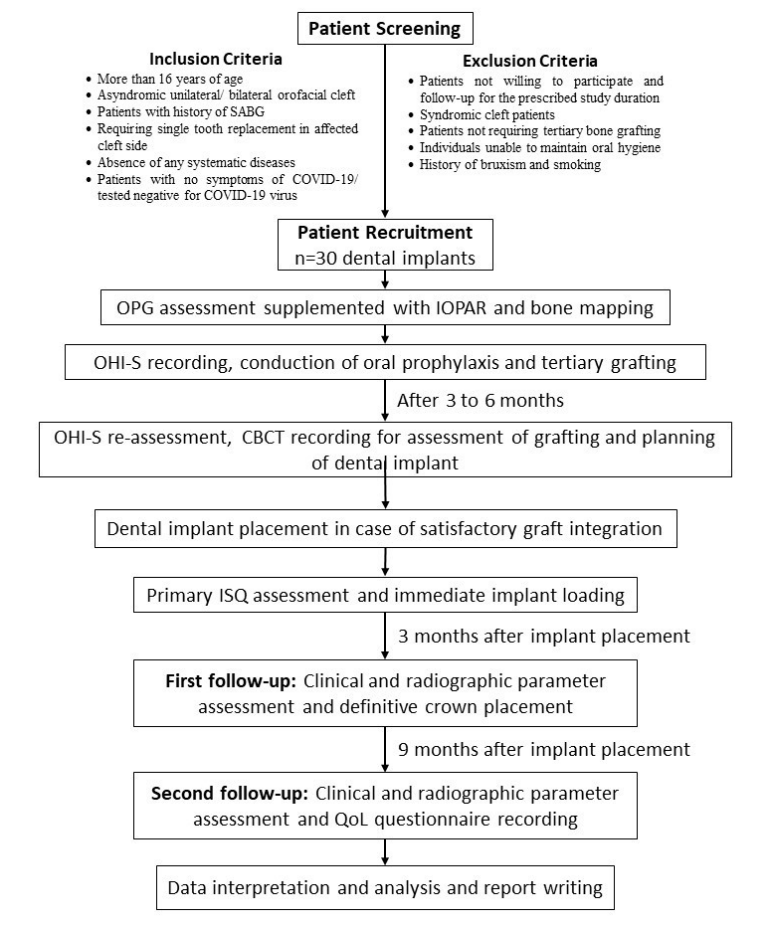
Summary of the research methodology. CBCT: cone beam computed tomography; IOPAR: intraoral periapical radiograph; ISQ: implant stability quotient; OHI-S: Oral Hygiene Index – Simplified; OPG: orthopantogram; QoL: quality of life; SABG: secondary alveolar bone grafting.

A single arm, prospective clinical trial evaluating the clinical and radiological success of immediately loaded dental implants in orofacial cleft patients will be conducted at the GSR Institute of CranioMaxillofacial & Facial Plastic Surgery, Hyderabad, Telangana, India. Ethical clearance for the clinical trial has been obtained from the Institutional Ethical Committees of the associated institutes. A summary of the methodology is presented in Figure 1.

### Sample Size Calculation

A recent study showed a success rate of 95% for dental implant–based treatment in cleft patients [10]. With a type I error of 5%, confidence interval of 95%, and 8% margin of error, a sample size of 28 dental implants was obtained. Considering 5% loss to follow-up, an additional 2 dental implants will be placed. Thus, a total sample size of 30 dental implants in sequential patients will be considered for the proposed study.

### Patient Selection

Without gender bias, consecutive patients meeting the inclusion and exclusion criteria will be recruited until a sample size of 30 dental implants is met. Signed informed consent to be part of the study will be obtained from the patient or their parent or guardian. Owing to the global COVID-19 pandemic, thorough COVID-19 history and consent will also be obtained. Institutional COVID-19 standard operating procedures will always be strictly followed. Before the beginning of treatment, each patient will be allotted a unique identification number to be used to designate them henceforth. Complete patient records including intra- and extra-oral photographs of the patients at all stages will be maintained, while maintaining confidentiality.

### Inclusion Criteria

To be included in the study, patients will need to be older than 16 years, have an asyndromic orofacial cleft, have a unilateral or bilateral cleft alveolus, have undergone secondary alveolar bone grafting (SABG) between 9 and 12 years of age, require single tooth replacement of the lateral incisor/canine region only on the affected cleft side, not have any systemic diseases, and have no symptoms of COVID-19 or tested negative for SARS-CoV-19 virus.

### Exclusion Criteria

Patients will be excluded when they are not willing to participate and follow-up for the prescribed study duration, have a syndromic cleft, do not require tertiary bone grafting, are unable to maintain oral hygiene (patients with a lack of manual dexterity or any kind of hand skeletal deformity), and have a history of bruxism and/or smoking.

### Operative Assessment

All patients with a single tooth missing in the cleft area will undergo preoperative screening using an orthopantogram (OPG) supplemented with an intraoral periapical radiograph (IOPAR) and bone mapping to determine the need for tertiary grafting. Prior to tertiary grafting, all patients will have the Oral Hygiene Index-Simplified (OHI-S) recorded followed by conduction of oral prophylaxis [11]. Following standard procedure guidelines, bone grafting will be done by a single operator using either an autologous symphyseal bone graft or iliac bone graft, depending on the amount of bone required [12-14]. Before proceeding with implant surgery, the OHI-S will be re-assessed and compared with the previously recorded observations. This will help to determine the patients’ attitudes towards oral health and their motivation towards maintaining oral hygiene. Cone beam computed tomography (CBCT) will be recorded 3-6 months after grafting to evaluate the integration of the grafted bone and plan implant placement. Before proceeding with implant surgery, the OHI-S will be re-assessed and compared with the previously recorded observations. In all patients, titanium dental implants (TitanGrade 4, blasted etched implant surface, Bredent GmbH Co, Senden, Germany) will be placed following a one-stage protocol. Additional bone graft material will be used to ensure complete bony coverage of the dental implant surface, if required. The implants will be placed slightly subcrestally, and the primary implant stability will be clinically evaluated by measuring implant stability quotient (ISQ) values using a Penguin^RFA^ device. The obtained values will be interpreted as follows [15]: ISQ ≥70: high initial stability and suitable for immediate loading; ISQ 55-70: moderate stability; ISQ ≤55: low/questionable stability and not suitable for immediate loading. Following the immediate loading protocol, all implants will be loaded with a provisional prosthesis made of autopolymerizing resin (ie, a provisional prosthesis will be placed within 7 days of dental implant placement) [16]. All patients will be educated about oral hygiene habits to ensure proper care of the placed implant and prosthesis along with use of a 0.12% chlorhexidine rinse for 30 seconds at least twice a day [17].

### Follow-Up

All patients will undergo 2 clinical follow ups, 3 months and 9 months after implant placement. At the first follow-up after a period of 3 months following immediate implant loading, the provisional prosthesis will be removed, and clinical and radiological parameters will be measured. Clinical parameters will include probing depth, bleeding on probing, suppuration, pain or tenderness in the implant area, and implant stability using ISQ values. The implant will then be loaded with a definitive prosthesis, and a CBCT will be recorded. A second follow-up will be performed after 6 months of definitive prosthesis placement (ie, a cumulative period of 9 months following implant placement), and all parameters will be assessed as stated previously. At the end of the treatment, all patients will be asked to complete a quality of life (QoL) assessment questionnaire to determine the patient’s perspective before and after dental implant treatment.

CBCT recordings will be done using a small sized field of view of approximately 50 mm in diameter, which has an effective dose of approximately 54µSv [18].

### Radiograph Interpretation

All recorded radiographs (OPG, IOPAR, and CBCT) will be assessed by 2 investigators. Both the investigators are trained professionals in the field of prosthodontics and oral and maxillofacial surgery, respectively, and have experience of more than 15 years in patient rehabilitation. Before beginning the study, both investigators will be trained in interpreting CBCTs of previously recorded cases that are not related to the current study to practice consistent reading. Each investigator will reassess the recorded radiographs after 1 month to determine intraobserver variability. The findings of the 2 investigators will also be subjected to evaluation of interobserver variability.

## Results

IBM SPSS version 23 and R 4.0.3 will be used for statistical analysis. Results will be aimed at determining the clinical and radiographic success of dental implants in patients with a nonsyndromic alveolar cleft. The Cox model of analysis will be used to determine the shared frailty of dental implants in case of bilateral cleft cases. The Kolmogrov-Smirnov test will be applied to determine the normality of the data. Depending on the data distribution, *t* tests, Wilcoxon tests, and chi-square tests will be utilized to determine statistical significance of clinical and radiological parameters assessed at 3 months and 9 months post implant placement. Binary logistic analysis will be done to compute the predictors of the outcome. CBCTs recorded after dental implant placement will be quantitatively compared to determine the presence of any significant variation in the marginal bone levels. Qualitative assessments of the radiographs will be done to determine the presence of periapical pathology and any other abnormal radiographic findings. Intra- and inter-observer variability in radiographic assessments will be evaluated by using the Kappa statistic. Statistical significance will be set at *P*<.05.

Implant success will be defined based on the guidelines given by Misch et al [19] in the Pisa, Italy Consensus and as presented in the patient record sheet. Statistical comments on the implant survival rate will be given by calculation of a life table analysis.

### Patient Record Sheet

#### Overview

Documentation of patient details forms an important part of diagnosis, treatment planning, and treatment outcomes. To avoid missing the recording of any details during a patient’s examination, it is good practice to have a preformed record sheet that is well thought through and encompasses all the required parameters. This also helps in standardization of the protocol, making future comparisons easier.

Dental implant–based rehabilitation is a precision-driven treatment that requires a careful pretreatment examination and investigations to determine the implant position and dimensions. Essential components of a record sheet for dental implant–based treatment have been highlighted and documented; however, no such standardization has been developed for cleft patients [20]. Thus, the presented patient record sheet is developed with the specific aim of implant-based rehabilitation of orofacial cleft patients (Multimedia Appendix 1). It incorporates patient demographics and clinical and follow-up findings essential for successful treatment outcomes, as described in the following sections.

#### Patient Demographics

The first part consists of essential patient details such as name, age, gender, contact details, and patient ID along with the type of cleft and its characteristics.

#### Past Surgical and Other Treatment History

This section helps to determine the patient’s previous medical history, associated complications, and any possible allergies. It also records the age of the patient at the time of SABG and the type of bone graft used. This forms an essential component of the record sheet as it helps in knowing the graft characteristics such as origin of the graft, time between grafting, and implant placement.

#### Prosthetic Considerations

This section focuses on intra-oral findings of the patient, highlighting the dental findings for the purpose of oral rehabilitation. This includes previous history of orthodontic treatment, age at which orthodontic treatment was done, missing teeth in the oral cavity, type of prosthesis previously used by the patient (if any), dentist’s and patient’s perceptions about the current prosthesis, and arch form characteristics.

#### Preoperative Assessment

This section records the findings concerning space of the edentulous ridge and corresponding OPG or CBCT findings.

#### Surgical Assessment

This section includes details concerning tertiary grafting and implant placement procedures.

#### Follow-Up Findings

In accordance with observations from previously conducted studies, important clinical and radiological parameters have been duly acknowledged in this section. This includes width of keratinized gingival, probing depths, and gingival and plaque index. It also records clinical findings such as bleeding on probing, suppuration, and pain or tenderness in the implant region. Recording of implant stability values using resonance frequency analysis (RFA) has been stressed since it is a noninvasive method and has shown good clinical results in healthy individuals. This is followed by recording of radiographic findings.

#### Implant Success

The last part enumerates the interpretation of the findings. Provision of this section avoids referring to multiple literature and provides a bird’s eye view of the important interpretations in a single frame [19].

### QoL Assessment Questionnaire

A QoL assessment questionnaire for the cleft population will be used to record their pre- and post-treatment experience with dental implant–based treatment.

## Discussion

Orofacial cleft is one of the most common developmental anomalies with a high global (1 in 700 to 1 in 1000 live births) and Indian (1.09 in 1000 live births) prevalence. In 1991, Verdi et al [21] were the first to employ the use of dental implants in cleft patients. In their findings, they stressed the need for cortical bone and adequate bone height in the required rehabilitation region for successful treatment. Since then, dental implants have been widely used for prosthetic rehabilitation of cleft patients with varying success rates (95.8% to 98.6%) [7,22,23]. In one of the biggest databases published by de Barros et al [24], the authors reported a high survival rate of 98.4% at the end of a 1-year follow-up period. Thus, dental implants provide a promising rehabilitative option for orofacial cleft patients.

All studies conducted so far have warranted a healing period of 3-6 months before undertaking the loading of the placed implant in the cleft site. The grafted bone in the cleft region is shown to have stable bone mineral densities during the period of 3-6 months following grafting, and thus, placing a dental implant in the grafted bone after 3 months of healing is considered adequate for successful treatment [25]. Until now, only 1 work published in 2011 has commented on the potential success of immediately loaded dental implants in orofacial cleft patients, but it was a retrospective analysis [8].

RFA is a relatively new and popular noninvasive technique for evaluating primary implant stability [15]. Due to the lack of immediate loading of dental implants in cleft patients, this technology still hasn’t found its application in such patients. Until now, the field of immediately loaded dental implants in cleft patients has been a barren land with no research conducted at the global level including India, despite the high prevalence of the deformity.

Success of dental implants is not only dependent on the achieved primary stability and subsequent osseointegration but also dictated by the soft tissue condition in the implant region. Cembranos et al [26], in their retrospective analysis of 47 implants, highlighted the importance of periodontal profile assessment along with measurements of bone levels to determine the success of implant-based treatment in cleft patients. Clinical and radiological evaluations of implant and adjacent sites in the form of probing depths, plaque and gingival indices, and marginal bone loss were also given importance in a similar analysis undertaken by Alberga et al [10].

Considering the global prevalence of deformity and lack of research in the field of immediately loaded dental implants in cleft patients, this study is formulated with an aim of evaluating the clinical and radiographic success of dental implant at the cleft site in orofacial cleft patients. At the same time, the proposed work also emphasizes the importance of having a preformed patient record sheet and need for recording various clinical and radiographic parameters and thus, has formulated a dentist-friendly and comprehensive record sheet that will be utilized in the study.

Apart from providing patients with enhanced functionality, replacement of the missing tooth will also enhance a patient’s aesthetics and their subsequent self-perception. During any rehabilitative procedure, it is important to know how the patient feels about it and what changes they see in their life pre- and post-treatment. This can be successfully evaluated using a QoL questionnaire. According to Burckhardt and Anderson [27], a QoL questionnaire encompasses details concerning material and physical well-being; relationships with other people; social, community, and civic activities; personal development and fulfilment; and recreation. QoL provides a meaningful way of determining the patient’s psychological improvement and making pre- and post-treatment comparisons.

### Scope

This study will help identify the success of immediately loaded dental implants for orofacial cleft patients and bring the same to regular clinical practice. Dental implants are a fixed treatment option that represent better functionality and aesthetics over conventional alternatives. Immediate loading of dental implants will not only prevent loss of the generated bone due to early functional stimulation but will also provide immediate aesthetic improvement, leading to an enhanced level of self-confidence of the patients. In cumulation, this will help increase patient’s and dentist’s acceptability of the treatment along with substantially reducing the treatment time and costs for such patients who have already undergone prolonged treatments since a young age.

### Limitations

Being a unique study, the proposed sample size is small compared to the huge prevalence of the deformity. Thus, studies with a larger sample size will be required in the future to give a statistically stronger result. Also, the study is not a case-controlled trial since the bone characteristics found in a cleft patient are difficult to replicate in a healthy individual. Any attempts to do so will lead to an increasing number of bias-related factors.

## Appendix

Multimedia Appendix 1 Patient record sheet.

## Abbreviations

CBCT: cone beam computed tomography
IOPAR: intraoral periapical radiograph
ISQ: implant stability quotient
OHI-S: Oral Hygiene Index - Simplified
OPG: orthopantogram
QoL: quality of life
RFA: resonance frequency analysis
SABG: secondary alveolar bone grafting
