# Supplementary material for: Assessment of Outcomes of Immediately Loaded Dental Implants in Orofacial Cleft Patients: Protocol for a Single-Arm Clinical Trial
Source: JMIR Res Protoc. 2021 May 5;10(5):e25244. doi: 10.2196/25244 (PMC8135029; doi:10.2196/25244)
Supplement: Multimedia Appendix 1 [file resprot_v10i5e25244_app1.docx]

**Patient Record Sheet**

**Patient ID:** __________________

**Patient’s Name:** _____________________ **Age/ Gender:** _____ years/ __________

**Address:** _________________________________________________________________________

**Contact no.:** ________________________

**Patient records given by**  Patient  Parent/ Guardian; Specify relation: _________

**Habits:**  Smoking  Bruxism  Tobacco Consumption  Others: _________________

**Type of cleft (select all that apply)**  Lip  Palate  Alveolus

**Characteristics of cleft**  Complete  Incomplete **Remarks, if any:** _________________________

☐ Unilateral ☐ Bilateral **If Unilateral Cleft** ☐ Right ☐ Left

**Past Surgical and Other Treatment History**

**Has patient been previously operated for any type of orofacial cleft repair?** Yes No

**(If yes, select one or more options below)**

Cleft lip  Cleft palate  Cleft lip and palate  Rhinoplasty

**Has the patient undergone secondary alveolar bone grafting?**  Yes  No

**If yes, age at secondary alveolar bone grafting *(with date of surgery, if known)*:** ___ years (_ _/_ _/_ _ _ _)

**Type of secondary bone graft used *(if known)*:** _____________________________________

**Any other relevant history:** ___________________________________________________________

**Prosthetic Consideration**

**Has the patient undergone orthodontic treatment?**  Yes  No

**If yes, at what age was the treatment undertaken?** ____________ years

**Circle the missing teeth**


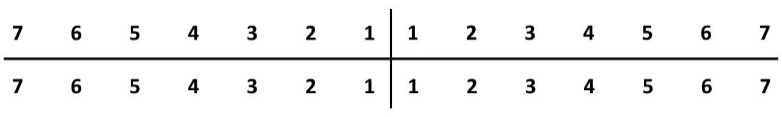


**Previous history of using any prosthesis for missing tooth in cleft region** ☐ Yes ☐ No

**If yes, type of prosthesis** __________________

**Current prosthesis evaluation (Dentist’s evaluation)**

**Stability and retention**  Poor  Average  Good

**Esthetics**  Poor  Average  Good

**Phonetics**  Poor  Average  Good

**Patient perception of current prosthesis**

**Stability and retention**  Poor  Average  Good

**Esthetics**  Poor  Average  Good

**Phonetics**  Poor  Average  Good

**Comfort**  Poor  Average  Good

**Arch form: Maxilla**  Square  Ovoid  Tapered

**Mandible**  Square  Ovoid  Tapered

**Any other relevant findings:** ______________________________________________________________

**Pre-operative Assessment**

**Space between teeth adjacent to edentulous cleft area:** _____ mm

**OPG/ CBCT findings**

**Dimensions of bone present in cleft area**

**Bone Height:** ______mm **Mesiodistal width:** ________mm

**Buccopalatal width:** ______mm **Bone Density:** ________Hu

**Presence of adequate bone for implant placement** ☐ Yes ☐ No

**Need for tertiary bone grafting on OPG assessment** ☐ Yes ☐ No

**Oral Hygiene Index – Simplified (OHI-S)**

**
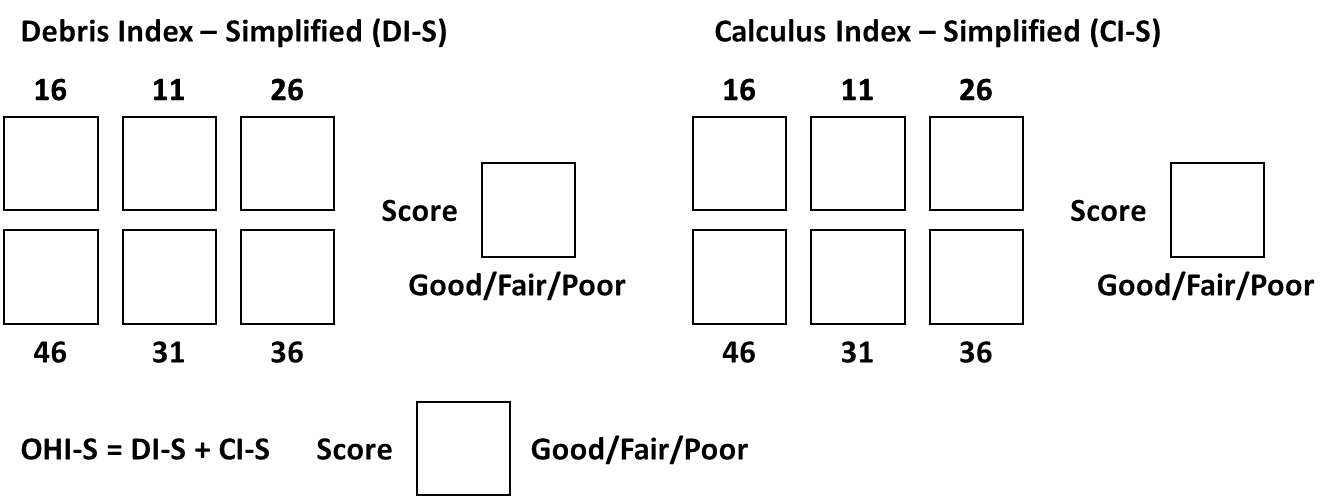
**

**Enemark Score**

| **Score** | **Normal bone height level** |  |
| --- | --- | --- |
| 1 | More than 75% |  |
| 2 | 50%-75% |  |
| 3 | 25%-50% |  |
| 4 | Less than 25% |  |

**
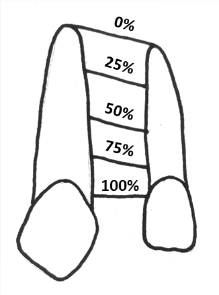
**

**Tertiary grafting**

**Date of Tertiary grafting:** ________________

**Graft used:** _____________________________________________

**Dimensions of the graft obtained:** _____________________________________________

**Additional material used:** _____________________________________________

**CBCT evaluation after tertiary grafting**

**Bone Height:** ______mm **Mesiodistal width:** ______mm

**Buccopalatal width:** ______mm **Bone Density:** ______Hu

**
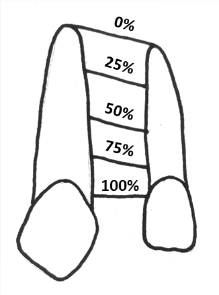
Enemark Score**

| **Score** | **Normal bone height level** |  |
| --- | --- | --- |
| 1 | More than 75% |  |
| 2 | 50%-75% |  |
| 3 | 25%-50% |  |
| 4 | Less than 25% |  |

**Implant Placement Surgery**

**Date of Implant Placement:** ________________

**Oral Hygiene Index – Simplified (OHI-S)**

**
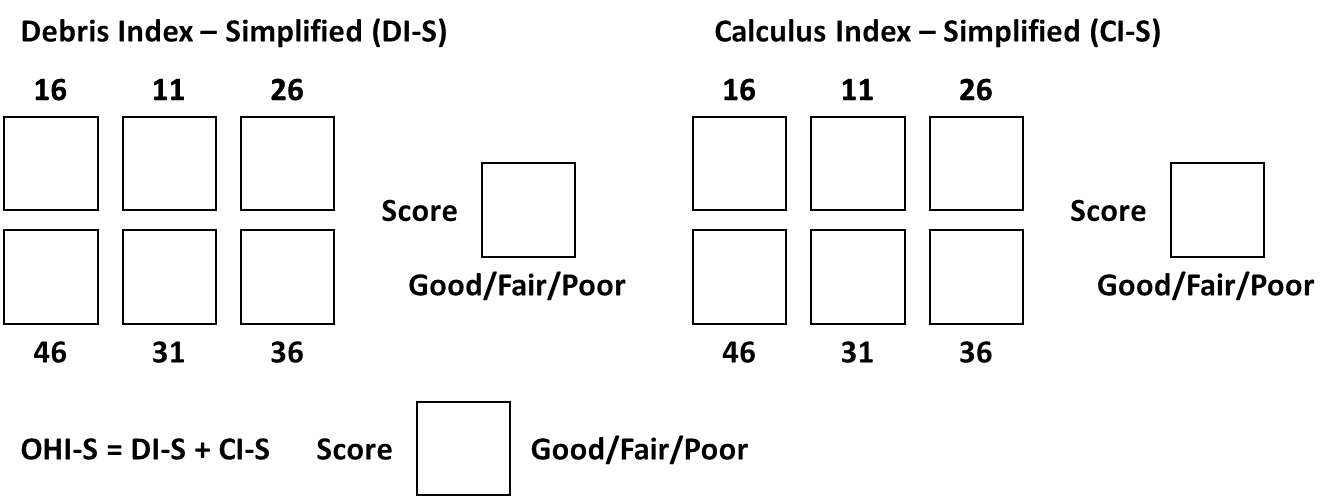
**

**Implant Specifications**

**Length:** _____ mm **Diameter:** _____ mm

**Insertion Torque:** _____ N/cm **Primary Implant Stability:** _____ ISQ

**Was immediate loading performed?**  Yes  No

**If yes, date of Immediate Loading:** _______________

**Any additional graft material was used during implant placement?**  Yes  No

**If yes, specify:** ___________________________________

**First follow-up (Definitive prosthesis placement)**

**Date:** _______________ **Done** ________ **months after dental implant placement**

**Width of Keratinized gingiva:** _____mm

| **Probing Depth** |  |  |  |  | **Gingival Index** |  |  |  |  | **Plaque Index** |  |  |  | |
| --- | --- | --- | --- | --- | --- | --- | --- | --- | --- | --- | --- | --- | --- | --- |
|  | **D** | **I** | **M** |  |  | **D** | **I** | **M** |  |  | **D** | **I** | **M** | |
| **D:** Tooth distal to the missing tooth; **I:** Missing tooth; **M:** Tooth mesial to the missing tooth | | | | | | | | | | | | | |  |

**Bleeding on probing:** ☐ Present ☐ Absent **Suppuration:** ☐ Present ☐ Absent

**Pain/ tenderness on palpation or percussion:** ☐ Present ☐ Absent

**Implant stability:** ________ ISQ

**Type of Definitive prosthesis:**  Cement retained  Screw Retained

**Material of definite prosthesis**:  PFM  All ceramic

Zirconia  Other (please specify): _____________

**Periapical Radiolucency**  Present  Absent

**Bone levels on CBCT: Mesial:** _____ mm **Distal:** _____ mm

**Buccal:** _____ mm **Palatal:** _____ mm

**9 months follow-up (1 year from the time of implant placement)**

**Date:** ________________

**Implant Stability:** _____ ISQ

**Width of Keratinized gingiva:** _____ mm

| **Probing Depth** |  |  |  |  | **Gingival Index** |  |  |  |  | **Plaque Index** |  |  |  | |
| --- | --- | --- | --- | --- | --- | --- | --- | --- | --- | --- | --- | --- | --- | --- |
|  | **D** | **I** | **M** |  |  | **D** | **I** | **M** |  |  | **D** | **I** | **M** | |
| **D:** Tooth distal to the missing tooth; **I:** Missing tooth; **M:** Tooth mesial to the missing tooth | | | | | | | | | | | | | |  |

**Bleeding on probing:** ☐ Present ☐ Absent

**Suppuration:** ☐ Present ☐ Absent

**Pain/ tenderness on palpation or percussion:** ☐ Present ☐ Absent

**Implant stability:** ________ ISQ

**Periapical Radiolucency**  Present  Absent

**Bone levels on CBCT: Mesial:** _____ mm **Distal:** _____ mm

**Buccal:** _____ mm **Palatal:** _____ mm

**Marginal Bone Loss since Definitive Implant Loading:** ________

**Implant success category**

|  | **Group I**  **Success/ Optimum Health** | **Group II**  **Satisfactory Survival** | **Group III**  **Compromised Survival** | **Group IV**  **Clinical/ Absolute Failure** |
| --- | --- | --- | --- | --- |
| **Pain/ Tenderness on palpation, percussion or function** | Absent | Absent | Absence of pain but sensitivity may be present | Present |
| **Mobility** | Absent | Absent | No vertical or initial horizontal mobility | Vertical and/ or horizontal mobility |
| **Radiographic Bone Loss** | <2mm | 2.0 - 4.0mm | >4mm crestal bone loss but <50% bone loss around implant (<½ of implant length) | Uncontrolled progressive bone loss with >50% bone loss around implant (>½ of implant length) |
| **Exudate/ Suppuration** | Absent | Absent | May or may not be present | Uncontrolled exudate |
| **Probing Depth** | No increase from baseline | No increase from baseline | Increased from baseline up to ½ the length of implant (>7mm);  BOP may or may not be present | Much more increase from baseline |
| **Prognosis** | Very good to excellent | Good to very good | Good to guarded | Failure |
|  |  |  |  |  |

**Legend: <:** Less than, **>:** More than, **g:** gram, **mm:** millimeter, **BOP:** Bleeding on Probing
